# Supplementary material for: A yeast phenomic model for the influence of Warburg metabolism on genetic buffering of doxorubicin
Source: Cancer Metab. 2019 Oct 23;7:9. doi: 10.1186/s40170-019-0201-3 (PMC6806529; doi:10.1186/s40170-019-0201-3)
Supplement: Supplementary file 9 — Additional file 9. GO term-specific heatmaps for REMc/GTF-enriched clusters. GO term-specific heatmaps for significant GO process terms were generated as described in methods and Figs. 3 and 4. Any related child terms are presented in subsequent pages of the parent file name. GO terms with more than 100 children, with 2 or fewer genes annotated to the term, or a file size over 300KB are not shown. All heatmaps are generated with the same layout (see Figs. 3 and 4). [file 40170_2019_201_MOESM9_ESM.bz2 › Additional_File9_GOTermHeatmaps/Additional_File9_GOTermHeatmap/protein_import.pdf]

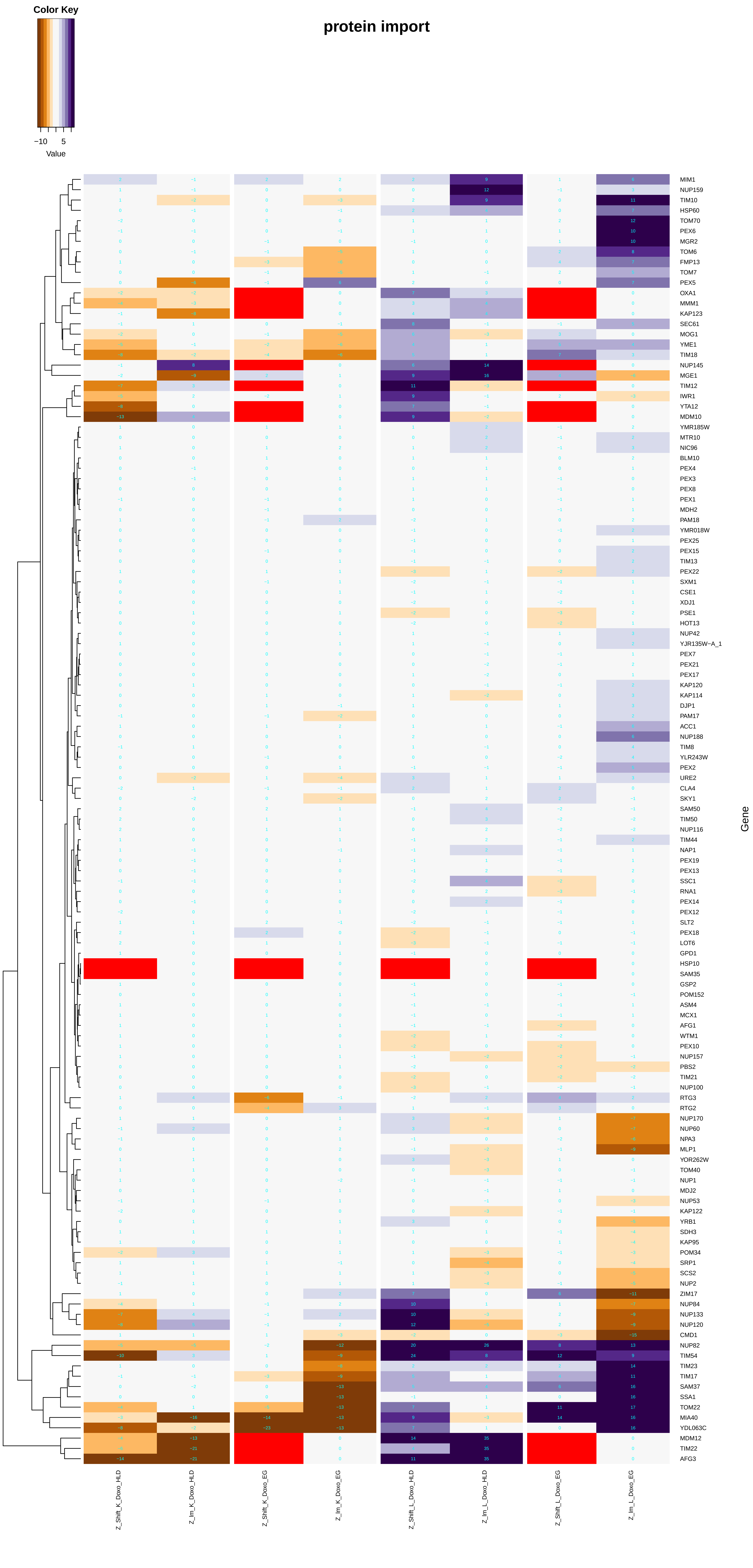
Color Key
−10
5
Value
protein import
MIM1
NUP159
TIM10
HSP60
TOM70
PEX6
MGR2
TOM6
FMP13
TOM7
PEX5
OXA1
MMM1
KAP123
SEC61
MOG1
YME1
TIM18
NUP145
MGE1
TIM12
IWR1
YTA12
MDM10
YMR185W
MTR10
NIC96
BLM10
PEX4
PEX3
PEX8
PEX1
MDH2
PAM18
YMR018W
PEX25
PEX15
TIM13
PEX22
SXM1
CSE1
XDJ1
PSE1
HOT13
NUP42
YJR135W−A_1
PEX7
PEX21
PEX17
KAP120
KAP114
DJP1
PAM17
ACC1
NUP188
TIM8
YLR243W
PEX2
URE2
CLA4
SKY1
SAM50
TIM50
NUP116
TIM44
NAP1
PEX19
PEX13
SSC1
RNA1
PEX14
PEX12
SLT2
PEX18
LOT6
GPD1
HSP10
SAM35
GSP2
POM152
ASM4
MCX1
AFG1
WTM1
PEX10
NUP157
PBS2
TIM21
NUP100
RTG3
RTG2
NUP170
NUP60
NPA3
MLP1
YOR262W
TOM40
NUP1
MDJ2
NUP53
KAP122
YRB1
SDH3
KAP95
POM34
SRP1
SCS2
NUP2
ZIM17
NUP84
NUP133
NUP120
CMD1
NUP82
TIM54
TIM23
TIM17
SAM37
SSA1
TOM22
MIA40
YDL063C
MDM12
TIM22
AFG3
Gene
Z_Shift_K_Doxo_HLD
Z_lm_K_Doxo_HLD
Z_Shift_K_Doxo_EG
Z_lm_K_Doxo_EG
Z_Shift_L_Doxo_HLD
Z_lm_L_Doxo_HLD
Z_Shift_L_Doxo_EG
Z_lm_L_Doxo_EG

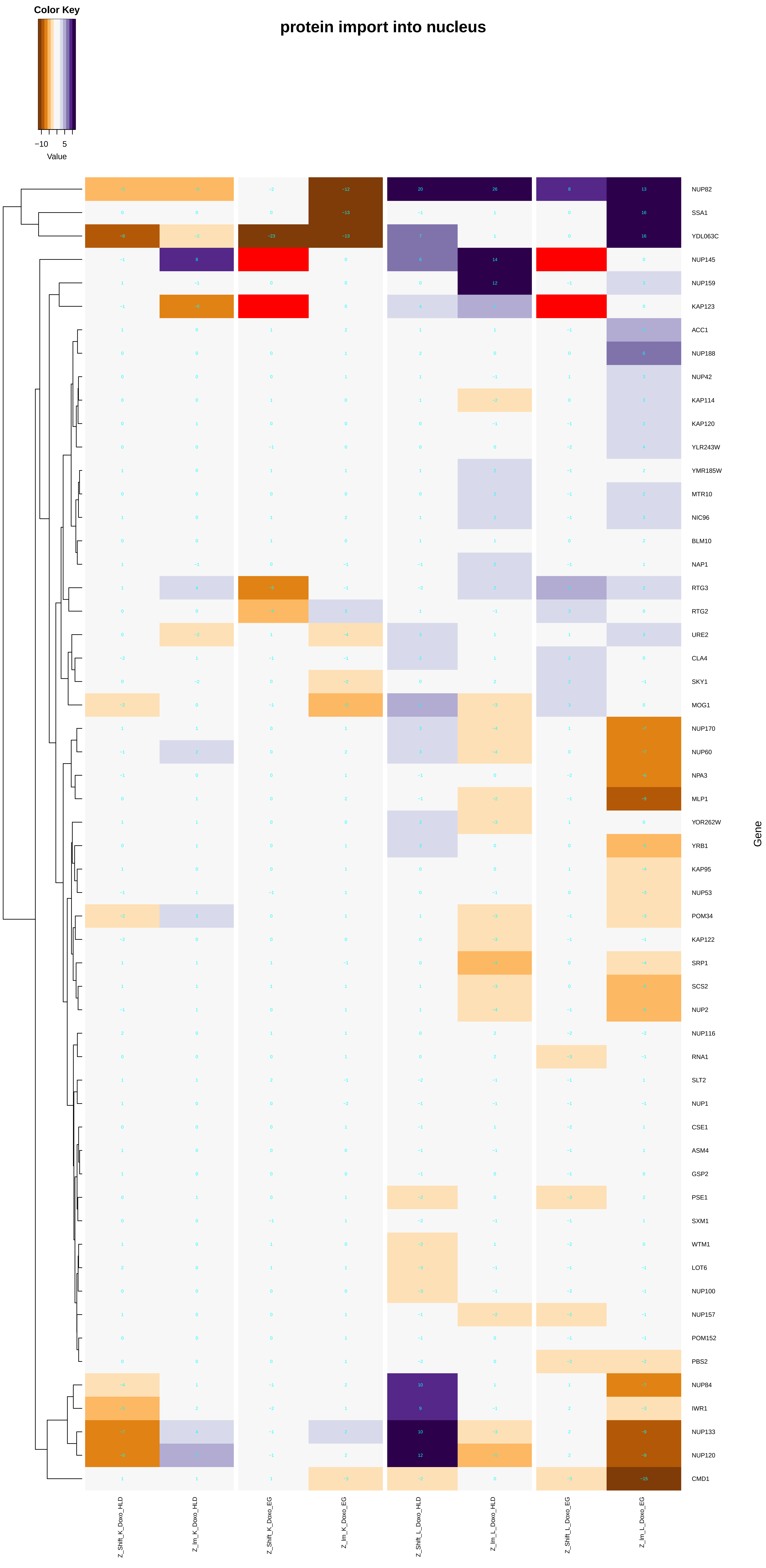
protein import into nucleus
Color Key
−10
5
Value
Gene
NUP82
SSA1
YDL063C
NUP145
NUP159
KAP123
ACC1
NUP188
NUP42
KAP114
KAP120
YLR243W
YMR185W
MTR10
NIC96
BLM10
NAP1
RTG3
RTG2
URE2
CLA4
SKY1
MOG1
NUP170
NUP60
NPA3
MLP1
YOR262W
YRB1
KAP95
NUP53
POM34
KAP122
SRP1
SCS2
NUP2
NUP116
RNA1
SLT2
NUP1
CSE1
ASM4
GSP2
PSE1
SXM1
WTM1
LOT6
NUP100
NUP157
POM152
PBS2
NUP84
IWR1
NUP133
NUP120
CMD1
Z_Shift_K_Doxo_HLD
Z_lm_K_Doxo_HLD
Z_Shift_K_Doxo_EG
Z_lm_K_Doxo_EG
Z_Shift_L_Doxo_HLD
Z_lm_L_Doxo_HLD
Z_Shift_L_Doxo_EG
Z_lm_L_Doxo_EG

intracellular protein transmembrane import

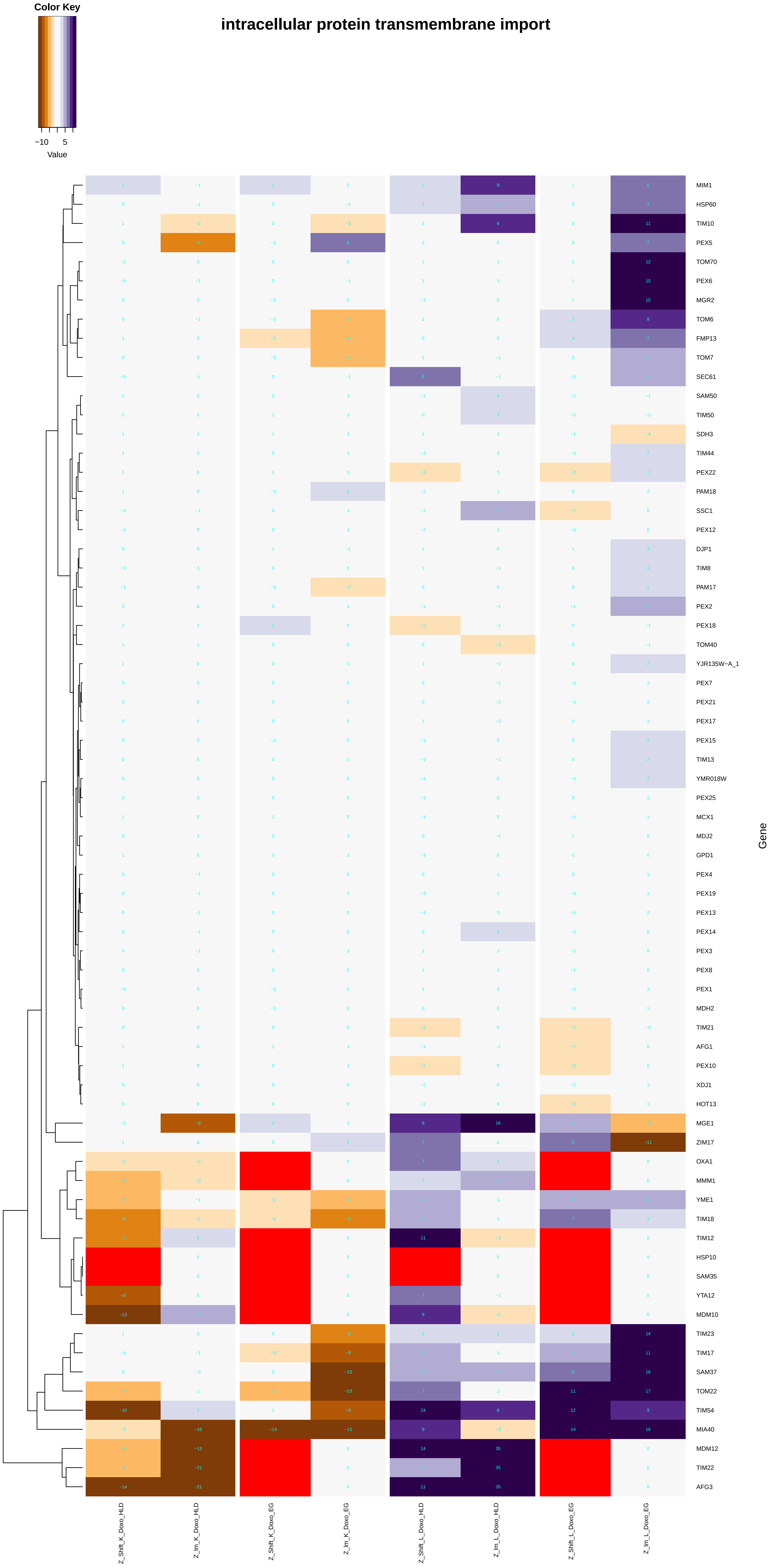

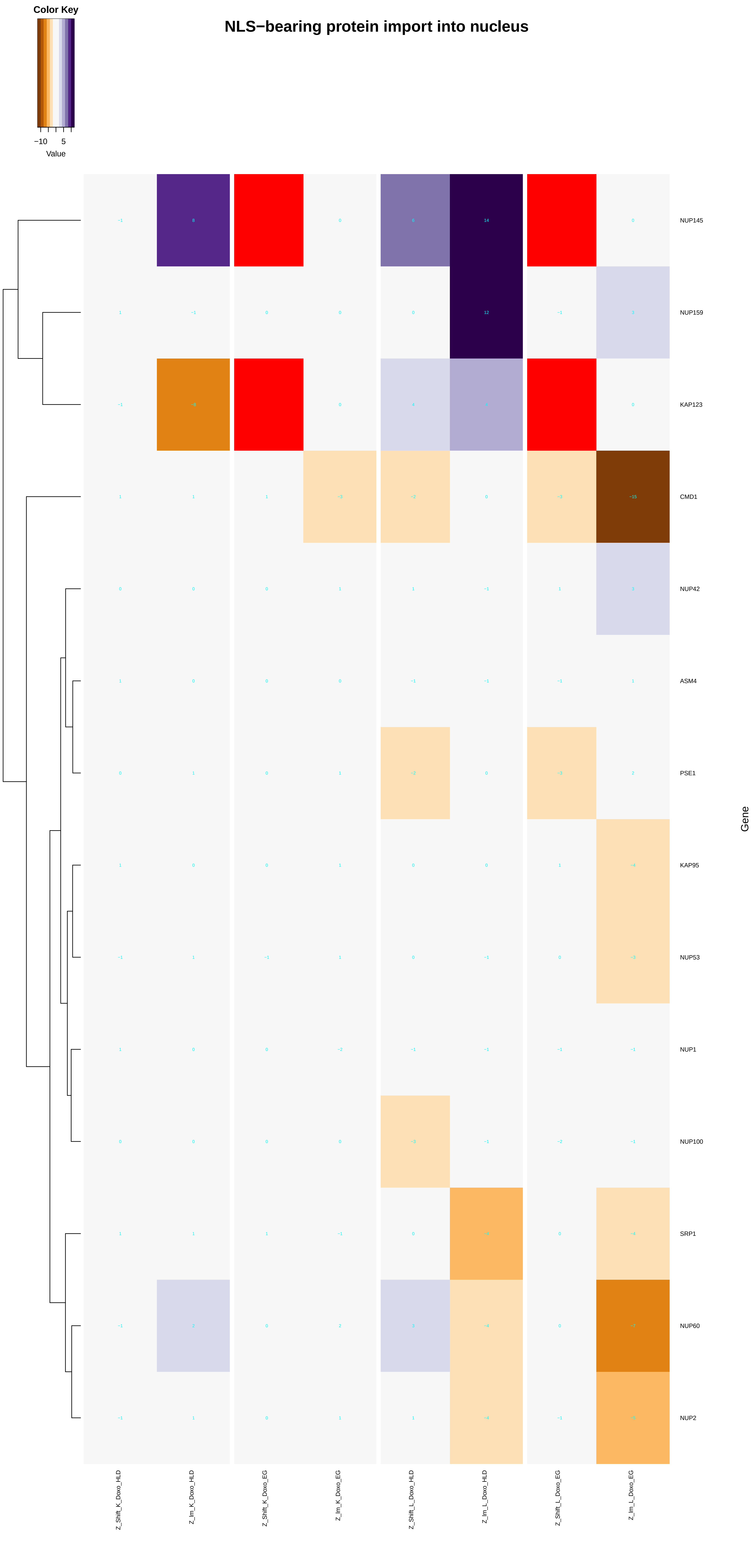
Color Key
−10
5
Value
NLS−bearing protein import into nucleus
NUP145
NUP159
KAP123
CMD1
NUP42
ASM4
PSE1
KAP95
NUP53
NUP1
NUP100
SRP1
NUP60
NUP2
Gene
Z_Shift_K_Doxo_HLD
Z_Im_K_Doxo_HLD
Z_Shift_K_Doxo_EG
Z_Im_K_Doxo_EG
Z_Shift_L_Doxo_HLD
Z_Im_L_Doxo_HLD
Z_Shift_L_Doxo_EG
Z_Im_L_Doxo_EG

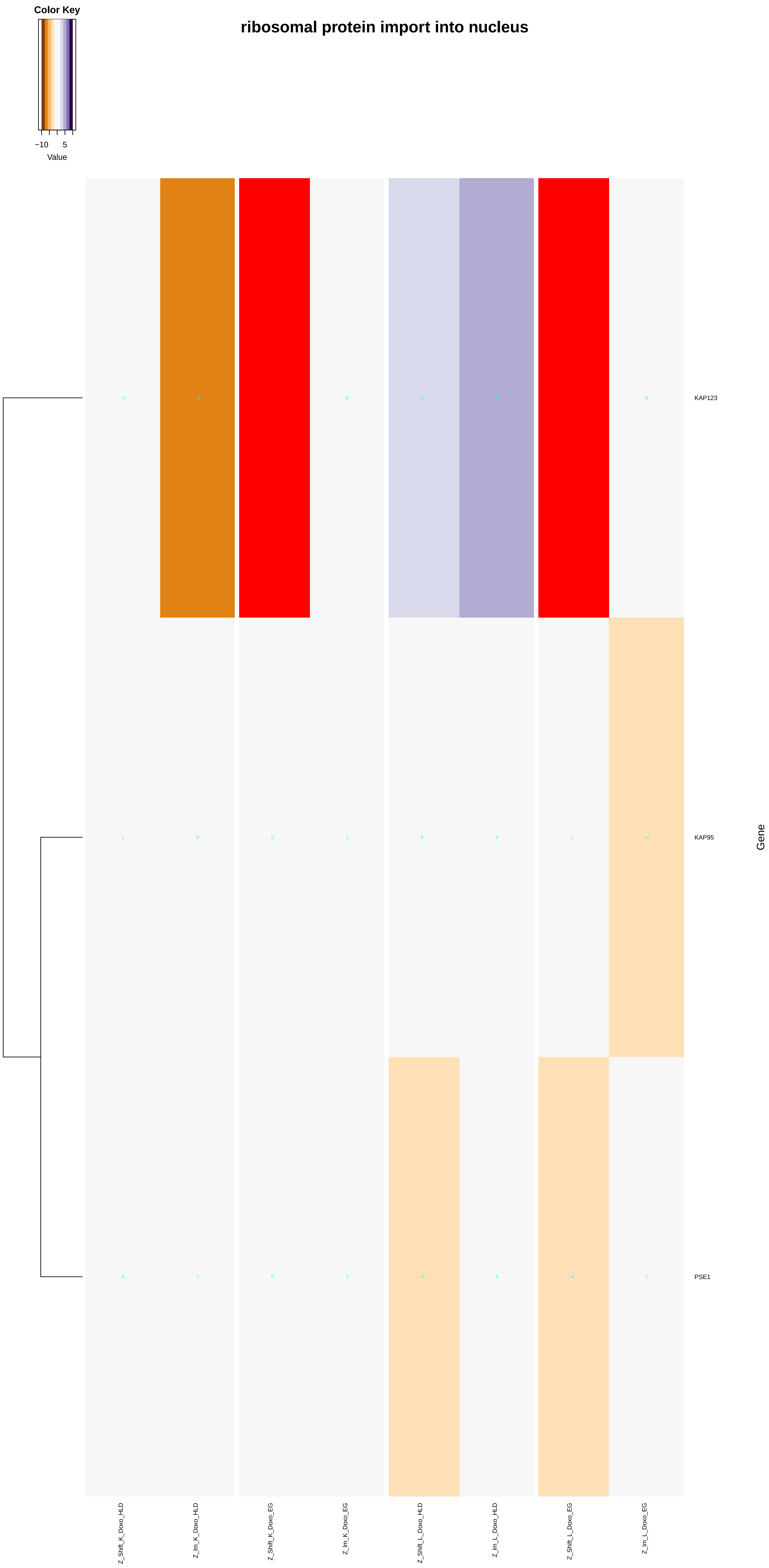

Color Key
−10
5
Value
ribosomal protein import into nucleus
KAP123
KAP95
PSE1
Gene
Z_Shift_K_Doxo_HLD
Z_Im_K_Doxo_HLD
Z_Shift_K_Doxo_EG
Z_Im_K_Doxo_EG
Z_Shift_L_Doxo_HLD
Z_Im_L_Doxo_HLD
Z_Shift_L_Doxo_EG
Z_Im_L_Doxo_EG

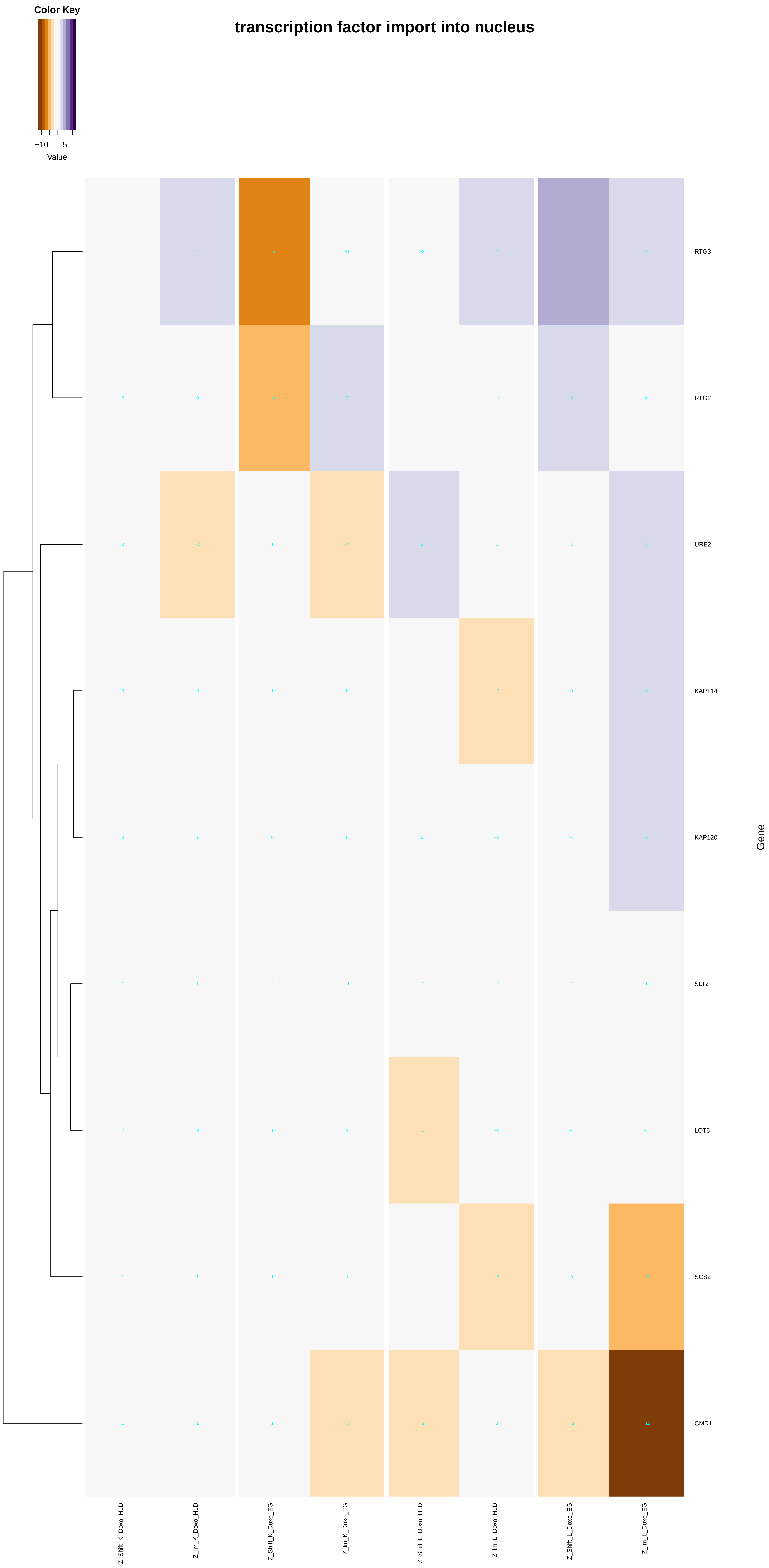
Color Key
−10
5
Value
transcription factor import into nucleus
RTG3
RTG2
URE2
KAP114
KAP120
SLT2
LOT6
SCS2
CMD1
Gene
Z_Shift_K_Doxo_HLD
Z_lm_K_Doxo_HLD
Z_Shift_K_Doxo_EG
Z_lm_K_Doxo_EG
Z_Shift_L_Doxo_HLD
Z_lm_L_Doxo_HLD
Z_Shift_L_Doxo_EG
Z_lm_L_Doxo_EG

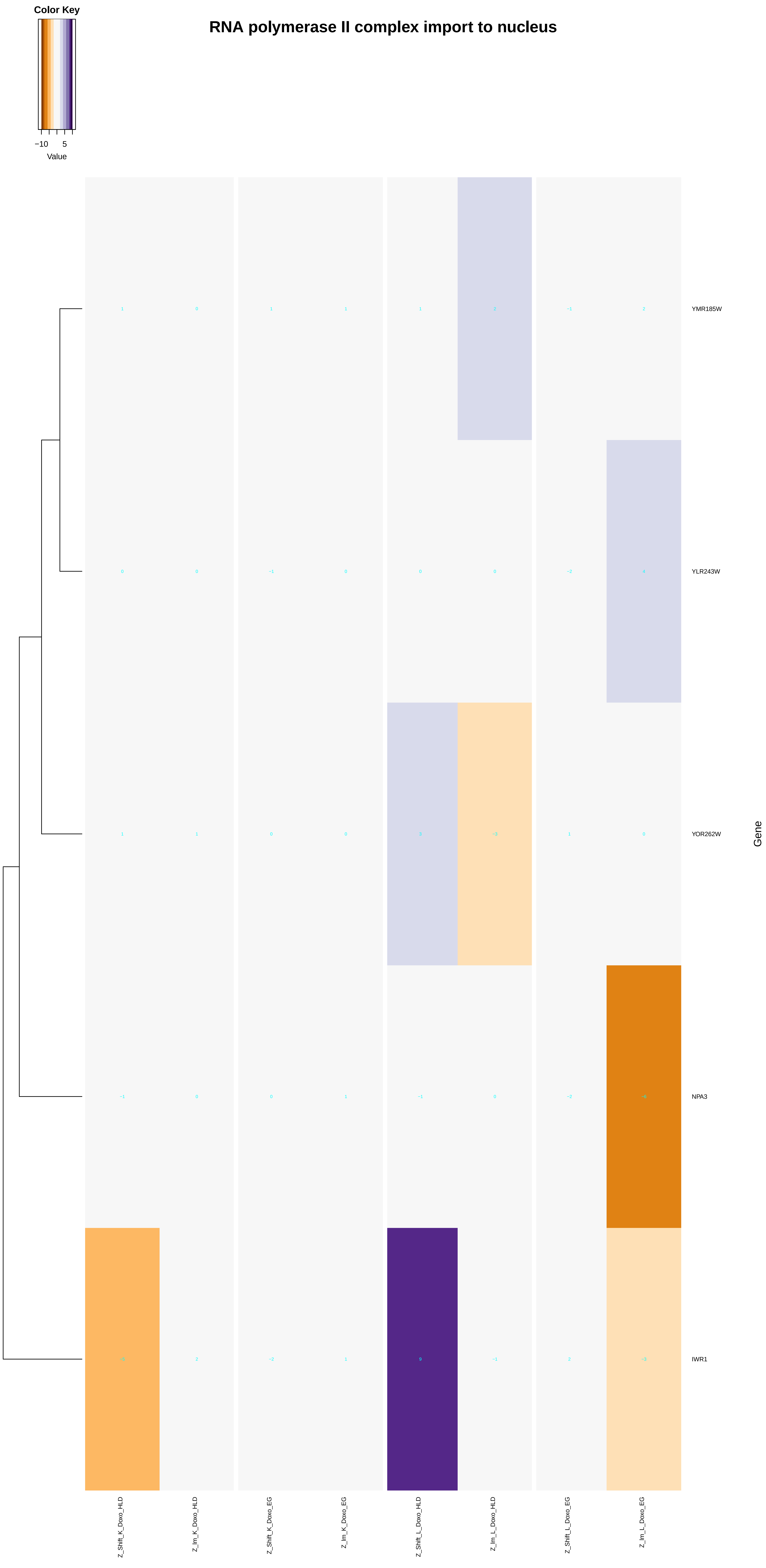

RNA polymerase II complex import to nucleus
Color Key
−10
5
Value
YMR185W
YLR243W
YOR262W
NPA3
IWR1
Gene
Z_Shift_K_Doxo_HLD
Z_Im_K_Doxo_HLD
Z_Shift_K_Doxo_EG
Z_Im_K_Doxo_EG
Z_Shift_L_Doxo_HLD
Z_Im_L_Doxo_HLD
Z_Shift_L_Doxo_EG
Z_Im_L_Doxo_EG

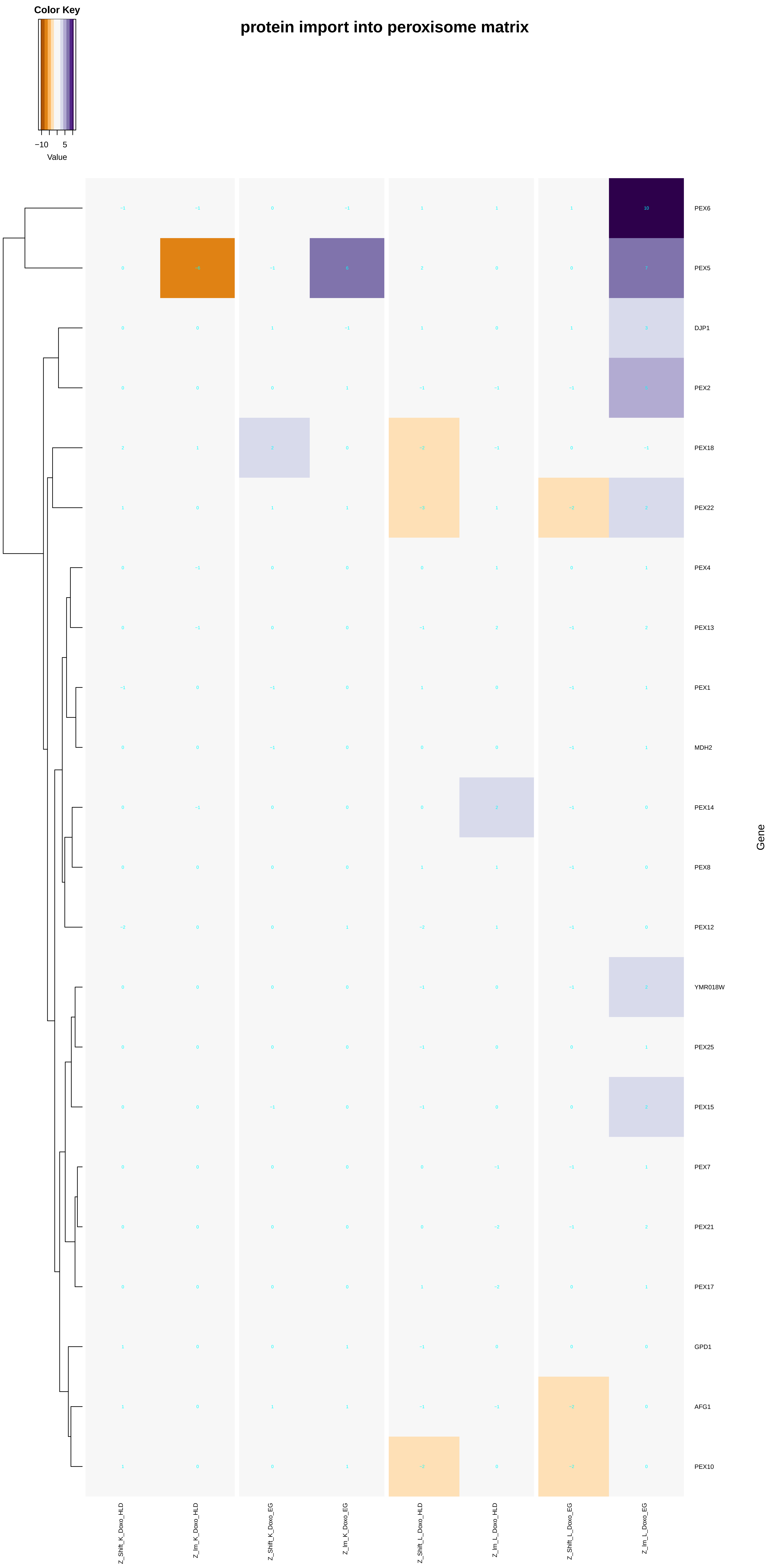
Color Key
−10
5
Value
protein import into peroxisome matrix
PEX6
PEX5
DJP1
PEX2
PEX18
PEX22
PEX4
PEX13
PEX1
MDH2
PEX14
PEX8
PEX12
YMR018W
PEX25
PEX15
PEX7
PEX21
PEX17
GPD1
AFG1
PEX10
Gene
Z_Shift_K_Doxo_HLD
Z_lm_K_Doxo_HLD
Z_Shift_K_Doxo_EG
Z_lm_K_Doxo_EG
Z_Shift_L_Doxo_HLD
Z_lm_L_Doxo_HLD
Z_Shift_L_Doxo_EG
Z_lm_L_Doxo_EG

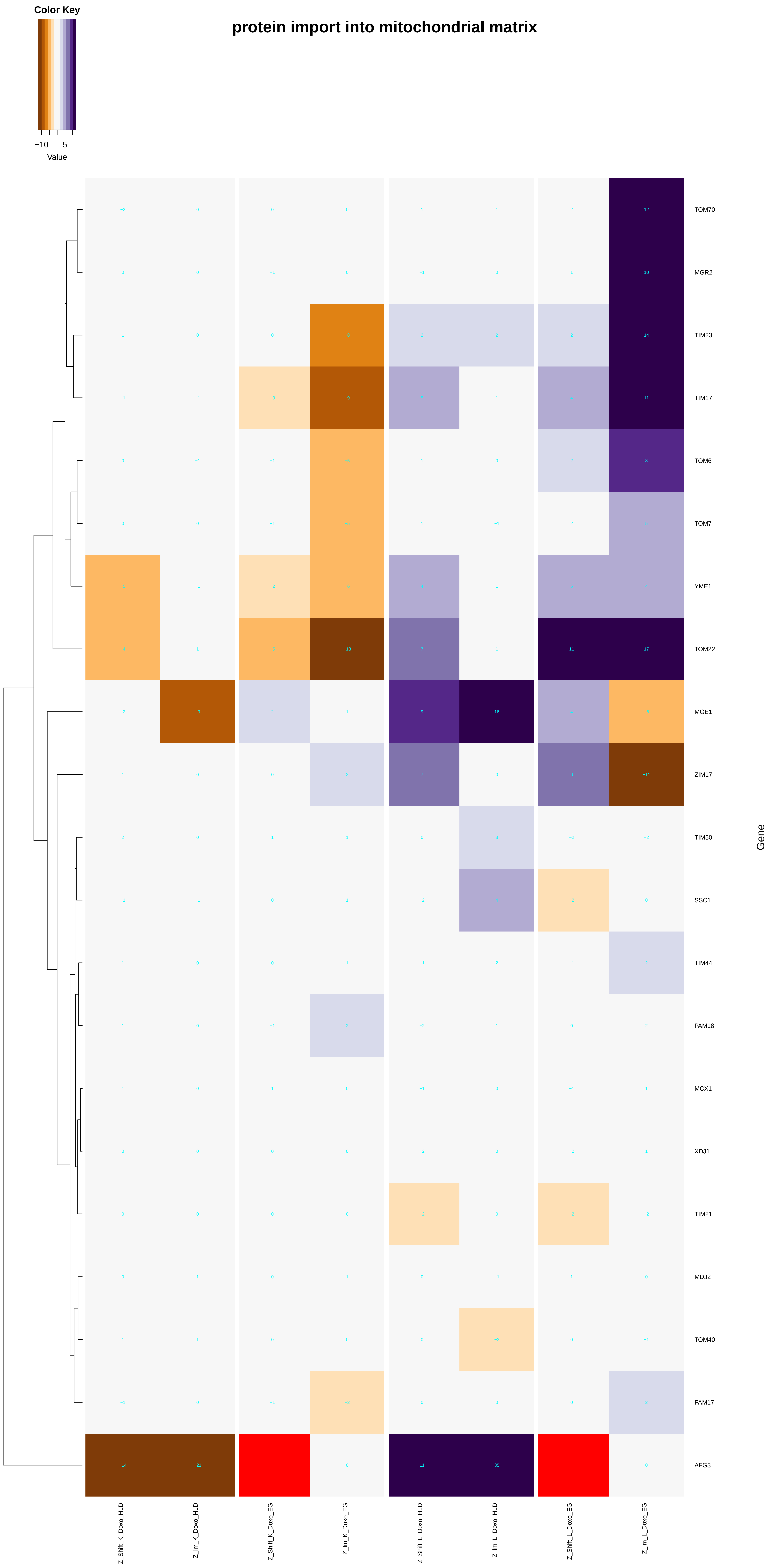
Color Key
−10
5
Value
protein import into mitochondrial matrix
TOM70
MGR2
TIM23
TIM17
TOM6
TOM7
YME1
TOM22
MGE1
ZIM17
TIM50
SSC1
TIM44
PAM18
MCX1
XDJ1
TIM21
MDJ2
TOM40
PAM17
AFG3
Gene
Z_Shift_K_Doxo_HLD
Z_Im_K_Doxo_HLD
Z_Shift_K_Doxo_EG
Z_Im_K_Doxo_EG
Z_Shift_L_Doxo_HLD
Z_Im_L_Doxo_HLD
Z_Shift_L_Doxo_EG
Z_Im_L_Doxo_EG

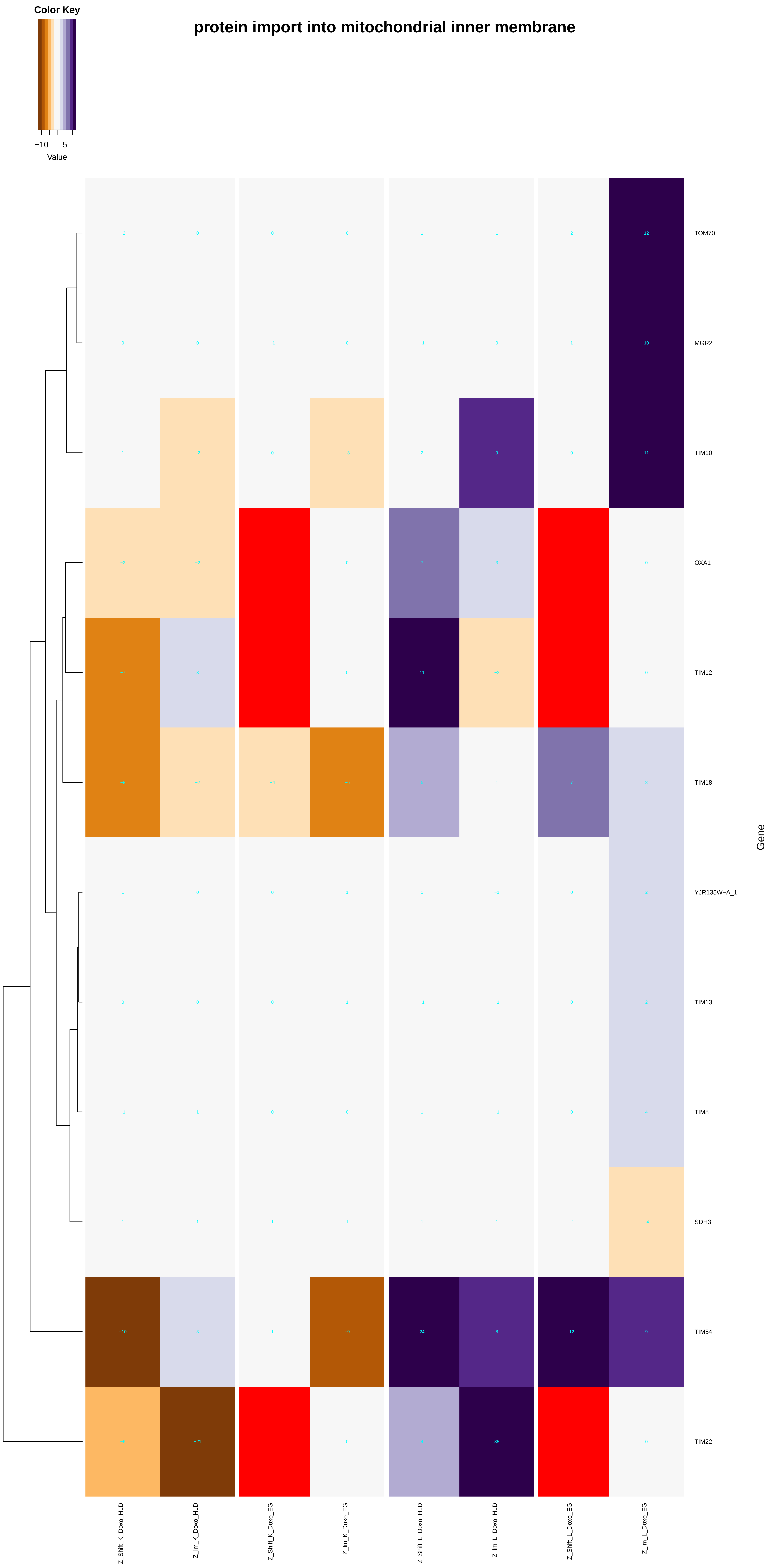
protein import into mitochondrial inner membrane
Color Key
−10
5
Value
TOM70
MGR2
TIM10
OXA1
TIM12
TIM18
YJR135W−A_1
TIM13
TIM8
SDH3
TIM54
TIM22
Gene
Z_Shift_K_Doxo_HLD
Z_Im_K_Doxo_HLD
Z_Shift_K_Doxo_EG
Z_Im_K_Doxo_EG
Z_Shift_L_Doxo_HLD
Z_Im_L_Doxo_HLD
Z_Shift_L_Doxo_EG
Z_Im_L_Doxo_EG

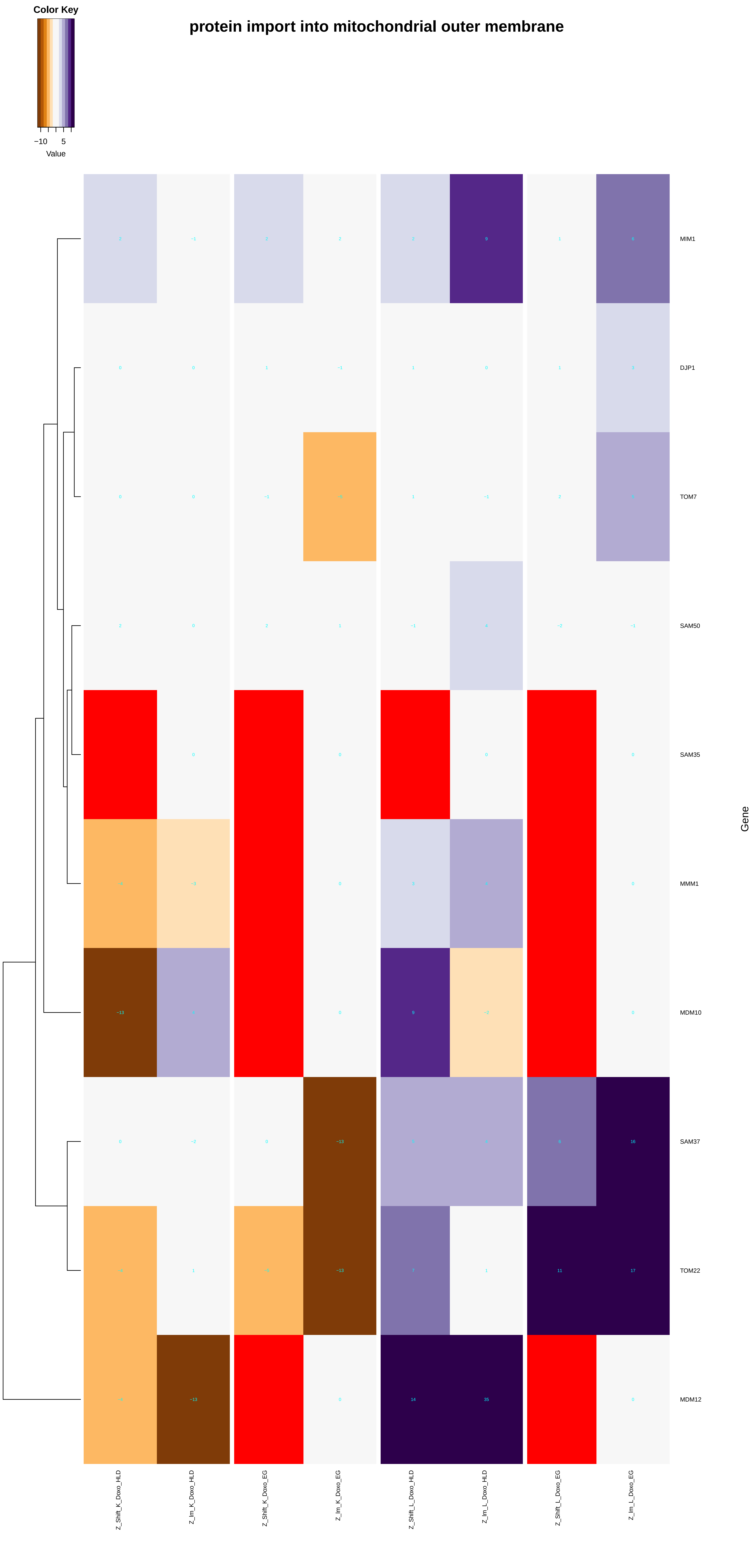

protein import into mitochondrial outer membrane
Color Key
−10
5
Value
MIM1
DJP1
TOM7
SAM50
SAM35
MMM1
MDM10
SAM37
TOM22
MDM12
Gene
Z_Shift_K_Doxo_HLD
Z_Im_K_Doxo_HLD
Z_Shift_K_Doxo_EG
Z_Im_K_Doxo_EG
Z_Shift_L_Doxo_HLD
Z_Im_L_Doxo_HLD
Z_Shift_L_Doxo_EG
Z_Im_L_Doxo_EG

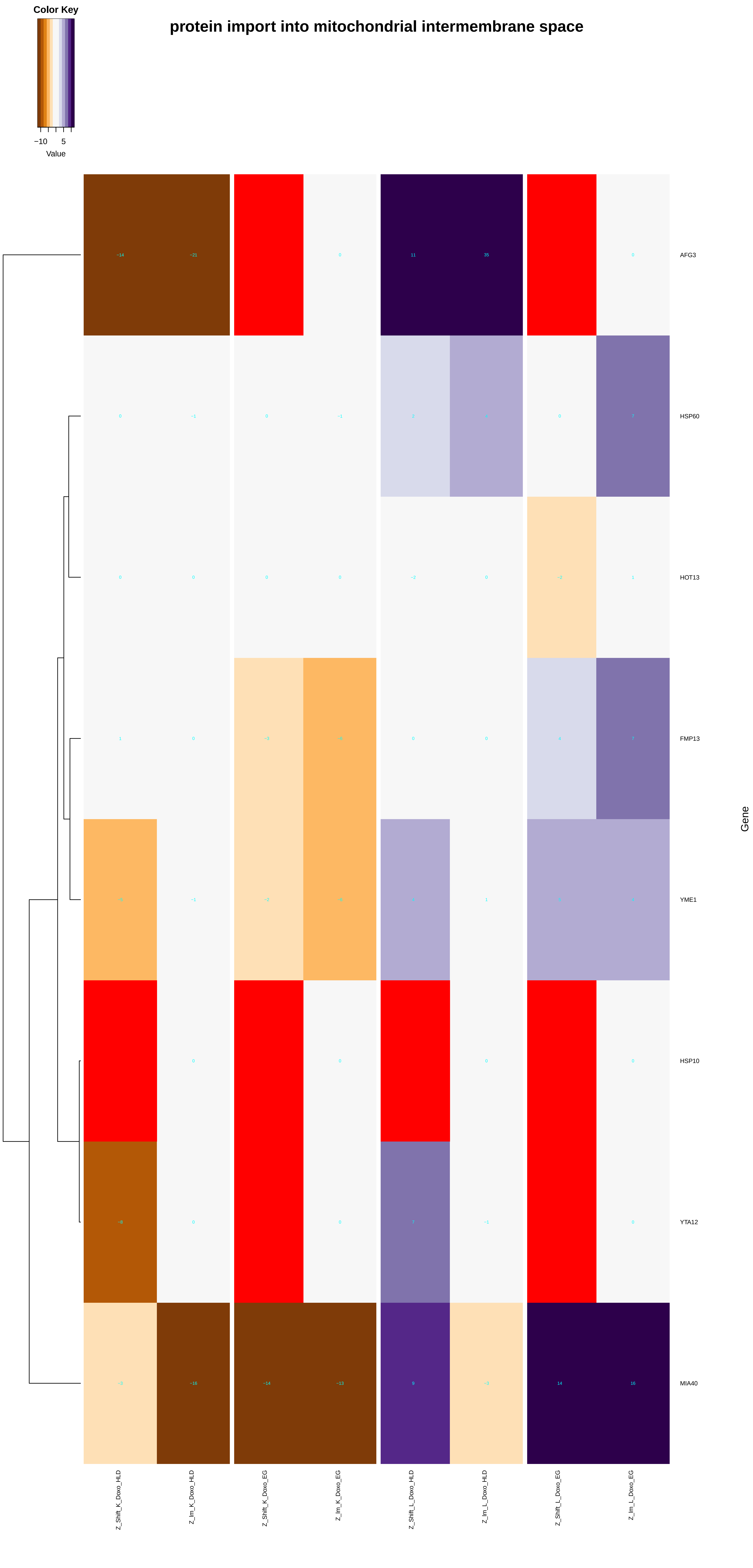

Color Key
−10
5
Value
protein import into mitochondrial intermembrane space
AFG3
HSP60
HOT13
FMP13
YME1
HSP10
YTA12
MIA40
Gene
Z_Shift_K_Doxo_HLD
Z_Im_K_Doxo_HLD
Z_Shift_K_Doxo_EG
Z_Im_K_Doxo_EG
Z_Shift_L_Doxo_HLD
Z_Im_L_Doxo_HLD
Z_Shift_L_Doxo_EG
Z_Im_L_Doxo_EG
